# Diagnostic Value of Concentration of Circulating Cell-Free DNA in Breast Cancer: A Meta-Analysis

**DOI:** 10.3389/fonc.2019.00095

**Published:** 2019-03-01

**Authors:** Dandan Yu, Yan Tong, Xinnian Guo, Lingfang Feng, Zhaoqiang Jiang, Shibo Ying, Junlin Jia, Yuan Fang, Min Yu, Hailing Xia, Li Shi, Jianlin Lou

**Affiliations:** ^1^Institute of Occupational Diseases, Zhejiang Academy of Medical Sciences (ZJAMS), Hangzhou, China; ^2^Department of Outpatient Office, Affiliated Hangzhou First People's Hospiital, Zhejiang University School of Medicine, Hangzhou, China

**Keywords:** circulating cell-free DNA, concentration, breast cancer, diagnostic value, meta-analysis

## Abstract

The diagnostic value of the concentration of circulating cell-free DNA (cfDNA) for breast cancer has generated inconsistent results. The aim of this study was to evaluate the first diagnostic value of the concentration of cfDNA for breast cancer by meta-analysis. Studies were retrieved by searching PubMed, Cochrane Library, and Web of Science before June 2018. Sensitivity, specificity, diagnostic odds ratio (DOR), the summary receiver operating characteristic (SROC) curve, and the area under curve (AUC) were used to summarize overall diagnostic performance. The random-effects model was used to calculate the pooled statistics. Subgroup analysis and meta-regression analysis were carried out to detect the source of heterogeneity. A total of 13 studies were identified with 1,087 breast cancer patients and 720 healthy controls. Overall, the pooled sensitivity and specificity of concentration of cfDNA for breast cancer were 87% (95% CI, 73–94%) and 87% (95% CI, 79–93%), respectively. The pooled DOR was 32.93 (95% CI, 13.52–80.19) and the SROC curve revealed an AUC of 0.93 (95% CI, 0.91–0.95). Meta-regression analysis showed that no covariate had a significant correlation with relative DOR (RDOR). Publication bias was not detected in this meta-analysis. This meta-analysis indicates that the concentration of cfDNA has potential first diagnostic value for breast cancer and plasma may be a better source of cfDNA for detection of breast cancer.

## Introduction

Breast cancer is one of the most common malignancies in women. According to an estimation from the WHO, more than 508,000 women worldwide died from breast cancer in 2011 (1). In China, breast cancer is the fourth most common cancer, with an estimated 150,000 cancer cases in 2012 (2). Early detection and diagnosis of breast cancer has been shown to reduce mortality (3). Currently, mammography is considered the gold standard in breast cancer early detection, which was also proven to be the only screening method to reduce mortality (4). However, mammography may fail to identify patients due to the overlapping dense fibroglandular tissue which reduce the visibility of tumor tissue or even entirely conceal the malignant lesions (5). It was reported that 15–30% of breast cancer was not detected while using full-field digital mammography (6). In addition, mammography may lead to over-diagnosis and radiation-induced disease. Therefore, in consideration of the limitation of mammography, it is necessary to develop a new non-invasive method to distinguish breast cancer patients from healthy individuals.

Cells can release DNA into the bloodstream, which is described as circulating cell-free DNA (cfDNA) (7). The cell-free DNA released from tumor cells, in particular, is called circulating tumor DNA (ctDNA) (7). Although cfDNA is also present in healthy individuals, it is significantly increased in cancer subjects (8). cfDNA is recognized as a novel biomarker in the diagnosis of cancer, such as gastric cancer (9), non-small lung cancer (10), and hepatocellular carcinoma (11). There are several detection strategies for breast cancer by cfDNA, such as the concentration of cfDNA, cfDNA integrity, microsatellite alteration, gene mutations, DNA methylation, and so on (12). The concentration of cfDNA is recognized as a quantitative way to detect cfDNA amounts and the initial detection strategy of breast cancer by cfDNA (12). So far, a number of studies have evaluated the diagnostic value of the concentration of cfDNA for breast cancer, but the results are inconsistent. For instance, Agostini et al. (13) reported a high sensitivity of 94.8% and a high specificity of 100%, while a study by Tang et al. (14) showed a low sensitivity of 65.0% and a low specificity of 70.0%. Hence, we conducted this meta-analysis to evaluate the first diagnostic value of the concentration of cfDNA for breast cancer.

## Materials and Methods

### Search Strategy

The following databases were searched to identify all potentially relevant studies published before June 2018: PubMed, Cochrane Library, and Web of Science. The retrieving query formulation used for the search were (“cell free DNA” OR “circulating DNA”OR “plasma DNA” OR “serum DNA”) AND (“breast cancer” OR “breast carcinoma” OR “breast tumor”). Article language was limited to English. All the reference lists of the identified articles and relevant reviews were also manually screened.

### Inclusion and Exclusion Criteria

The inclusion criteria of eligible studies were as follows: (1) studies about the first diagnosis not the recurrent diagnosis of the concentration of cfDNA for breast cancer; (2) studies with sufficient data for describing or calculating the sensitivity and specificity values; and (3) studies that were case-control studies, prospective, and retrospective cohort studies. The exclusion criteria included: (1) studies that were reviews, case-only studies, conference letters, or editorials and (2) studies with duplicate data reported.

### Data Extraction and Quality Assessment

Two reviewers (Dandan Yu and Yan Tong) screened titles, abstracts, and full texts independently according to the above criteria. The following data were extracted from enrolled studies in structured forms: first author's name, publication year, country, sample size, study type, source of cfDNA, time of sample collection, test method, reference gene, cut-off value, sensitivity, and specificity. Subsequently, two reviewers independently evaluated the quality of selected studies according to Quality Assessment of Diagnostic Accuracy Studies-2 (QUADAS-2) (15).

### Statistical Analysis

The pooled statistics with 95% confidence intervals (95% CIs) of sensitivity, specificity, and diagnostic odds ratio (DOR) were calculated. The summary receiver operating characteristic (SROC) curve and the area under curve (AUC) were used to summarize overall diagnostic performance. Additionally, we used the χ^2^ test and inconsistency index (*I*^2^) to quantify the statistical heterogeneity between studies. A *P* < 0.1 by χ^2^ test and *I*^2^ statistic >50% indicated substantial heterogeneity (16). A fixed-effects model was used to calculate the pooled statistics if there was no statistical heterogeneity; otherwise, a random-effects model was conducted. Furthermore, subgroup analysis and meta-regression analysis were carried out to detect the source of heterogeneity. Publication bias was evaluated with Deeks' funnel plot asymmetry test (17). *P* < 0.05 indicated potential publication bias.

All statistical analysis was performed with STATA (version 14.0; Stata Corp, College Station, TX). A *P* < 0.05 was considered statistically significant, and all *P*-values were two-sided.

## Results

### Study Characteristics and Quality Assessment

**Figure 1:**
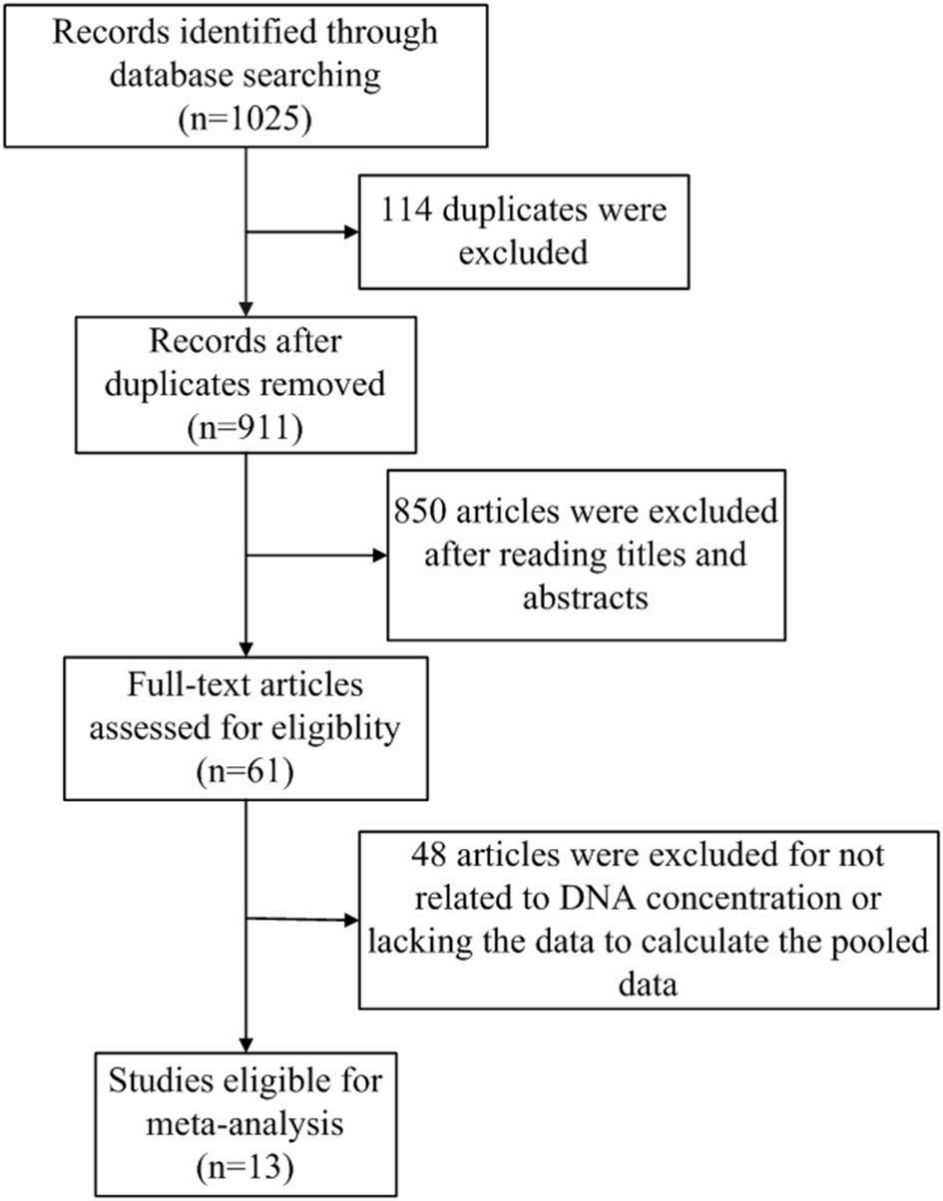
Flow diagram of the study selection process.

The flowchart of literature search and selection were shown in Figure 1. A total of 1,025 records were initially identified by our search strategy. Ultimately, 13 studies (13, 14, 18–28) were eligible for this meta-analysis according to the inclusion criteria and the exclusion criteria. A total of 1,807 subjects including 1,087 breast cancer patients and 720 healthy controls were recruited for analysis. The sensitivity and specificity of cfDNA for breast cancer detection in selected studies ranged from 37.5 to 100% and from 57.1 to 100%, respectively. Characteristics of these studies are summarized in Table 1.

**Table 1:** Characteristics of studies included in this meta-analysis of concentration of circulating cell-free DNA in the diagnosis of breast cancer.

**References**	**Country**	**No. of P/C**	**Study type**	**Sample**	**Time of sample collection**	**Test method**	**Reference gene**	**Cut of value**	**Sensitivity (%)**	**Specificity (%)**
Leon et al. (22)	USA	32/55	Case-control	Serum	BS	Radioimmu-noassay	–	50 ng/ml	37.5	92.7
Gal et al. (24)	UK	96/24	Case-control	Serum	BS	RT-qPCR	β-globin	–	70.8	93.7
Huang et al. (18)	China	61/27	Cohort	Plasma	BS	RT-qPCR	β-globin	19 ng/ml	95.1	88.9
Catarino et al. (25)	Portugal	175/80	Cohort	Plasma	AS	RT-qPCR	hTERT	106.0 ng/mL	43.4	91.2
Kohler et al. (19)	Switzerland	52/70	Cohort	Plasma	BS	RT-qPCR	GAPDH	1866 GE/ml	81.0	69.0
Agostini et al. (13)	Italy	39/49	Case-control	Plasma	BS	RT-qPCR	ALU115	9.3394 ng/ml	94.8	100.0
Hashad et al. (20)	Egypt	42/27	Case-control	Plasma	BS	RT-qPCR	hTERT	34 ng/ml	97.6	96.3
Gong et al. (21)	China	200/100	Cohort	Serum	BS	RT-qPCR	GAPDH	471 ng/ml	94.0	95.0
Stötzer et al. (26)	Germany	112/28	Case-control	Plasma	BS	RT-qPCR	ALU115	–	95.0	57.1
Agassi et al. (23)	Israel	38/16	Cohort	Serum	BS	Fluorescent SYBR Gold stain	–	600 ng/mL	72.0	75.0
Tangvarasittichai et al. (27)	Thailand	100/100	Case-control	Plasma	BS	Qubit^TM^flu-orometer	–	120 ng/ml	100.0	88.6
Zhang et al. (28)	China	100/104	Case-control	Serum	BS	RT-qPCR	HOTAIR	Relative concent-ration 0.30	80.0	68.3
Tang et al. (14)	China	40/40	Case-control	Serum	–	Fluorescence qPCR	ALU115	300.96 ng/mL	65.0	70.0

*P, patients; C, controls; BS, before surgery; AS, after surgery; RT-qPCR, real-time quantitative PCR*.

The quality of selected studies by QUADAS-2 is shown in Table 2. To some extent, most studies had a moderate-high quality, the overall quality of these included studies were generally robust.

**Table 2:** Assessment of the methodological quality by QUADAS-2.

**References**	**Risk of bias**				**Applicability concerns**		
	**Patient selection**	**Index test**	**Reference standard**	**Flow and timing**	**Patient selection**	**Index test**	**Reference standard**
Leon et al. (22)	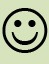	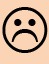	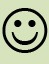	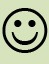	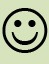	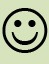	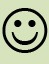
Gal et al. (24)	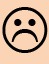	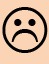	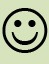	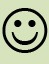	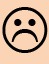	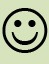	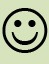
Huang et al. (18)	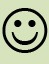	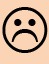	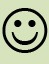	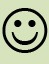	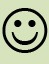	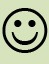	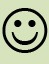
Catarino et al. (25)	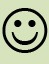	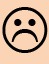	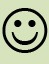	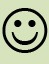	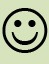	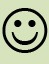	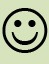
Kohler et al. (19)	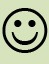	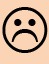	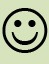	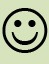	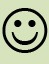	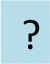	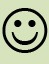
Agostini et al. (13)	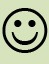	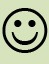	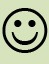	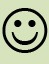	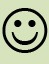	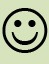	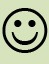
Hashad et al. (20)	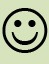	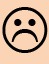	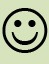	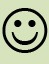	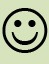	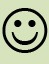	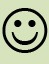
Gong et al. (21)	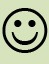	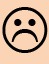	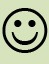	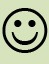	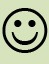	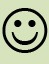	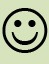
Stötzer et al. (26)	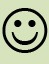	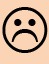	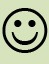	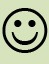	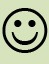	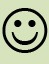	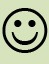
Agassi et al. (23)	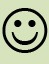	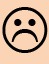	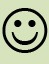	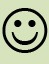	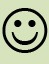	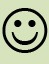	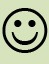
Tangvarasittichai et al. (27)	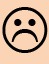	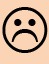	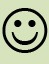	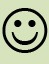	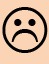	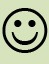	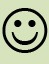
Zhang et al. (28)	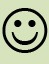	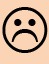	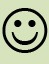	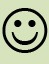	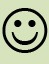	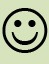	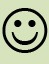
Tang et al. (14)	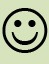	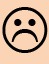	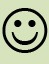	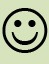	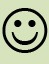	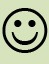	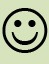

*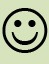, low risk*;

*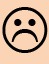, high rsk*;

*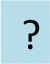, unclear risk*.

### Diagnostic Value of Concentration of cfDNA for Breast Cancer

**Figure 2:**
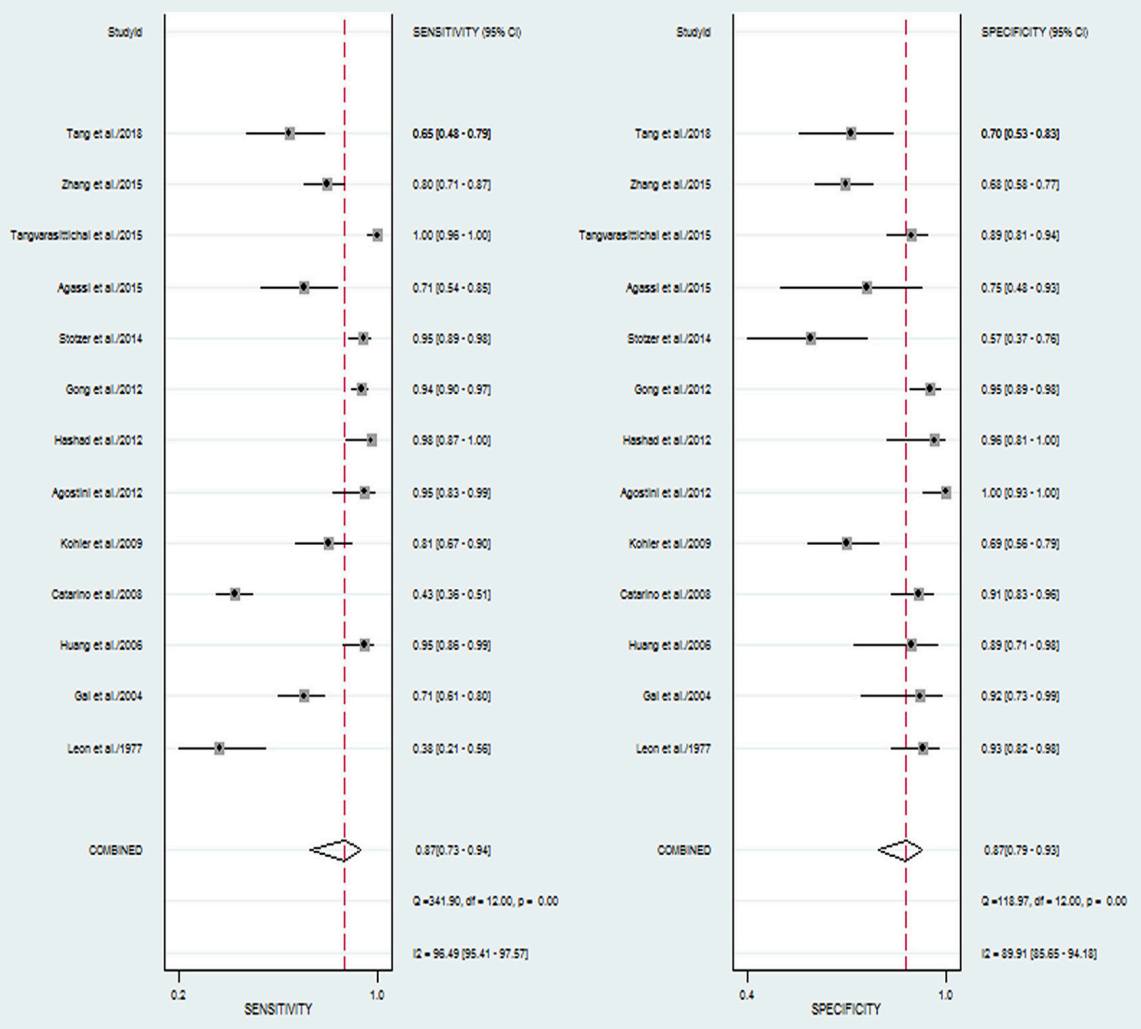
Forest plots of sensitivity and specificity for diagnostic value of concentration of circulating cell-free DNA for breast cancer.

**Figure 3:**
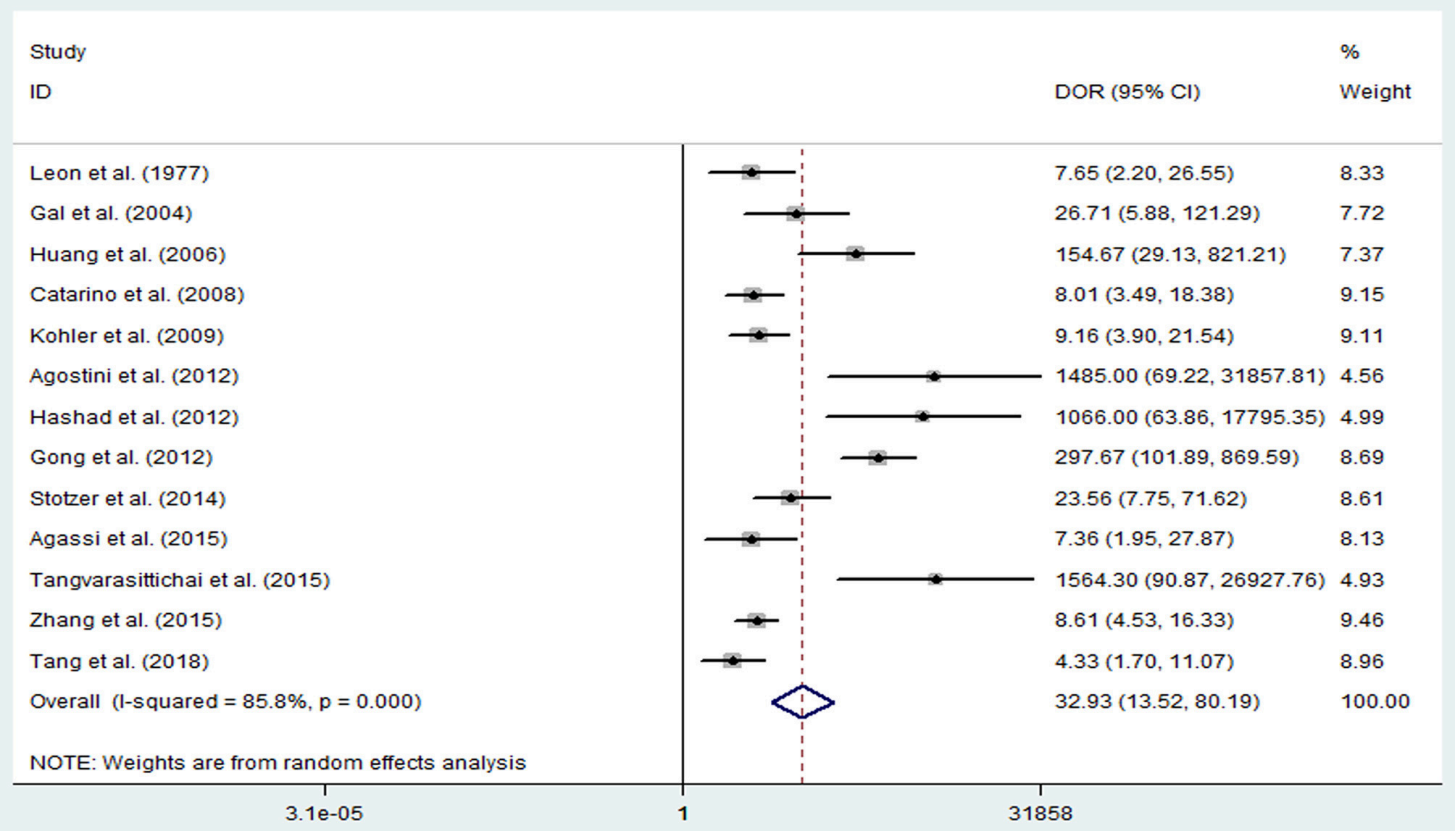
Diagnostic odds ratio of sensitivity and specificity for diagnostic value of concentration of circulating cell-free DNA for breast cancer.

**Figure 4:**
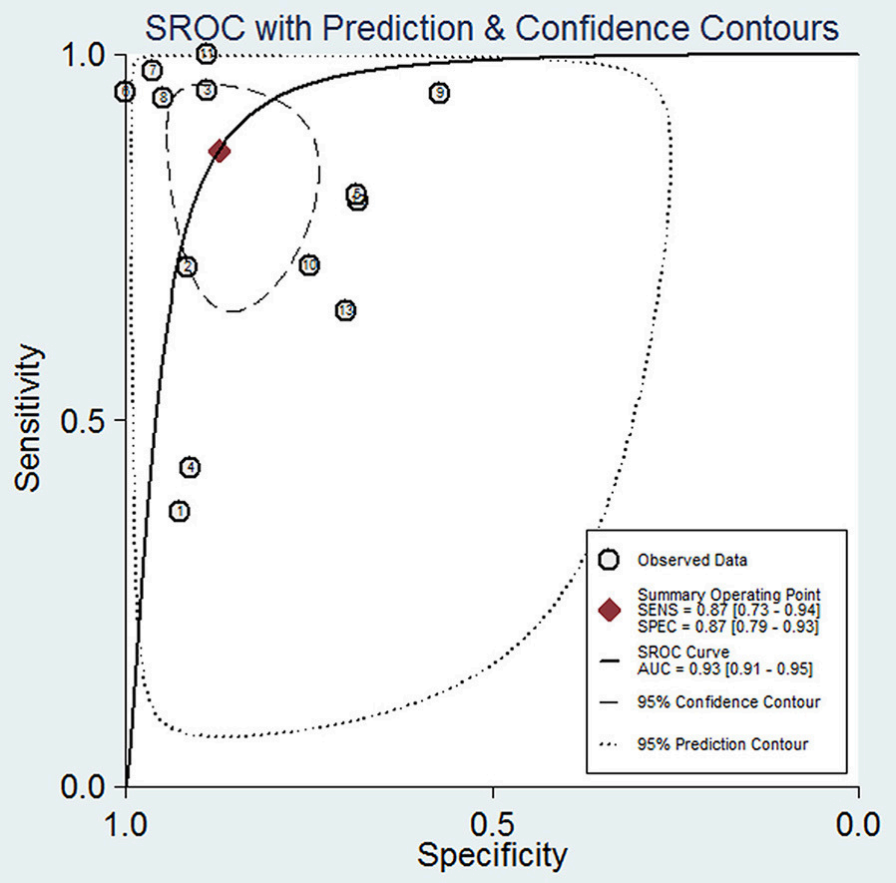
Summary receiver operating characteristic of sensitivity and specificity for diagnostic value of concentration of circulating cell-free DNA for breast cancer.

The pooled sensitivity and specificity of concentration of cfDNA for breast cancer were 87% (95% CI, 73–94%) and 87% (95% CI, 79–93%), respectively (Figure 2, Table 3). The pooled DOR was 32.93 (95% CI, 13.52–80.19) (Figure 3, Table 3). The SROC curve revealed an AUC of 0.93 (95% CI, 0.91–0.95) (Figure 4, Table 3).

**Table 3:** Summary of main results of diagnostic value of concentration of cfDNA for breast cancer.

**Study group**		***N***	**Sensitivity (95% CI)**	***I*^2^ (%)**	**Specificity (95% CI)**	***I*^2^ (%)**	**DOR (95% CI)**	***I*^2^ (%)**	**AUC (95% CI)**
All		13	87% (73%−94%)	96.49	87% (79%−93%)	89.91	32.93(13.52–80.19)	85.8	0.93(0.91–0.95)
Sample of cfDNA	plasma	7	94% (80%−98%)	98.52	89% (75%−96%)	94.26	79.11(19.13–327.12)	85.0	0.97(0.95–0.98)
	serum	6	74% (56%−86%)	92.54	85% (73%−92%)	85.77	15.80 (4.51–55.34)	88.3	0.87(0.84–0.90)
Time of sample collection	before surgery	11	90% (78%−96%)	94.1	88% (78%−94%)	89.17	50.15(17.49–143.79)	86.1	0.95(0.93–0.96)
Test method	RT–qPCR	9	88% (77%−95%)	97.52	89% (77%−95%)	93.66	46.29(15.82–135.45)	86.8	0.95(0.93–0.96)
	Not RT–qPCR	4	85% (29%−99%)	95.04	84% (73%−91%)	74.64	16.60 (2.62–105.31)	85.0	0.88(0.85–0.91)

*N, number of studies*.

Sample of cfDNA, the time of sample collection, and test method were taken into consideration for subgroup analysis. With consideration of the sample of cfDNA, studies with cfDNA isolated from plasma revealed that the pooled sensitivity and specificity were 94% (95% CI, 80–98%) and 89% (95% CI, 75–96%), respectively (Table 3). The pooled DOR was 79.11 (95% CI, 19.13–327.12), and the SROC curve revealed an AUC of 0.97 (95%CI, 0.95–0.98) (Table 3). Studies with cfDNA isolated from serum revealed that the pooled sensitivity and specificity were 74% (95% CI, 56–86%) and 85% (95% CI, 73–92%), respectively (Table 3). The pooled DOR was 15.80 (95% CI, 4.51–55.34), and the SROC curve revealed an AUC of 0.87 (95%CI, 0.84–0.90) (Table 3).

With consideration of the time of sample collection, studies with samples collected before surgery revealed that the pooled sensitivity and specificity were 90% (95% CI, 78–96%) and 88% (95% CI, 78–94%), respectively (Table 3). The pooled DOR was 50.15 (95% CI, 17.49–143.79), and the SROC curve revealed an AUC of 0.95 (95% CI, 0.93–0.96) (Table 3).

With consideration of the test method, studies with the test method of real-time qPCR revealed that the pooled sensitivity and specificity were 88% (95% CI, 77–95%) and 89% (95% CI, 77–95%), respectively (Table 3). The pooled DOR was 46.29 (95% CI, 15.82–135.45), and the SROC curve revealed an AUC of 0.95 (95% CI, 0.93–0.96) (Table 3). Studies without the test method of real-time qPCR showed that the pooled sensitivity and specificity were 85% (95% CI, 29–99%) and 84% (95% CI, 73–91%), respectively (Table 3). The pooled DOR was 16.60 (95% CI, 2.62–105.31), and the SROC curve revealed an AUC of 0.88 (95% CI, 0.85–0.91) (Table 3).

### Meta-Regression Analysis and Publication Bias

**Figure 5:**
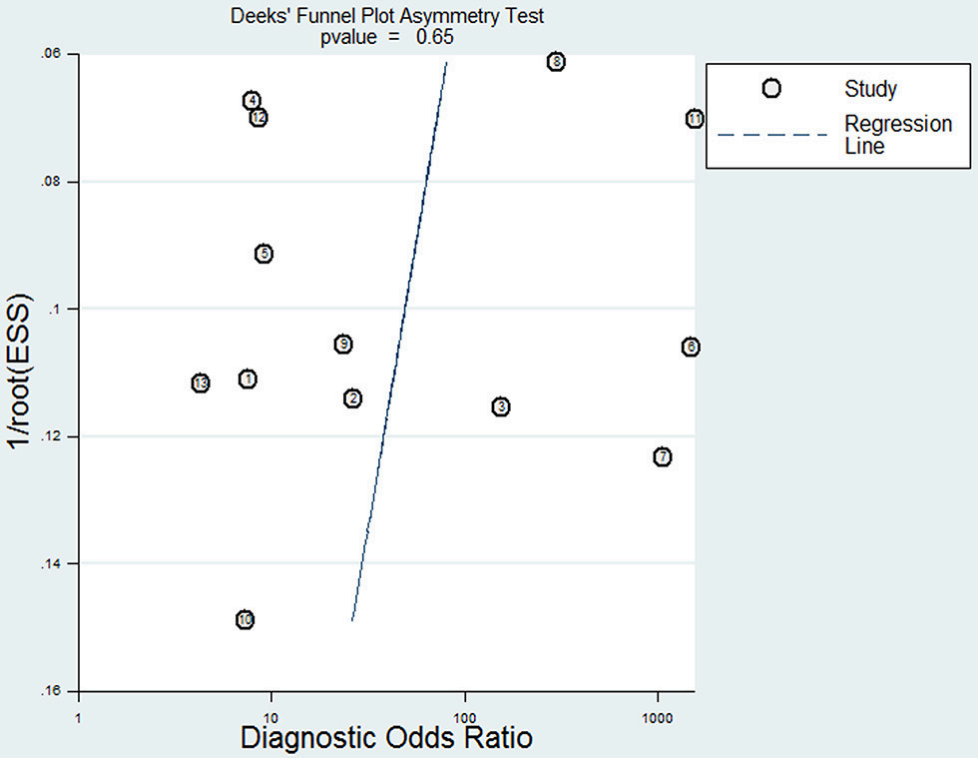
Deeks' funnel plot for the assessment of potential publication bias in this meta-analysis.

To reveal the sources of heterogeneity, we conducted a meta-regression analysis. Covariates including “publication year (recent 5 years),” “region (Asian),” “sample (plasma),” “time of sample collection (before surgery),” and “method (real-time quantitative PCR)” were assessed. We used these covariates to assess their effects on the RDOR. None of them showed any definite influence on heterogeneity and the details of the calculation were showed in Table 4. Deeks' funnel plot asymmetry test was used to assess publication bias. The *P*-value was 0.65, which showed there was no significant evidence of publication bias (Figure 5).

**Table 4:** Meta-regression of effects of study characteristics on diagnostic accuracy of concentration of cfDNA.

**Covariates**	**Coefficient**	**SE**	***P***	**RDOR (95% CI)**
Year	1.526	6.252	0.299	4.598 (0.184–114.463)
Region	−1.862	0.207	0.206	0.155 (0.007–3.662)
Sample	−2.179	0.142	0.127	0.113 (0.006–2.212)
Time of sample collection	−2.133	0.171	0.182	0.118 (0.004–3.576)
Test method	0.283	1.888	0.848	1.328 (0.046–38.349)

*SE, standard error*.

## Discussion

As is known, mammography as a screening method for breast cancer has the limitation of having low sensitivity. Hence, the circulating biomarkers, which can be easily collected and measured, have attracted the attention of more researchers. A correlation between increased levels of cfDNA and cancer has been widely studied (29, 30). Nowadays, it has been widely accepted that a solid tumor will obviously increase cfDNA concentrations (31). It was also demonstrated that the circulating cfDNA concentrations of breast cancer patients were significantly higher than healthy controls (25).

During the past decades, biomarkers of breast cancer have been widely discovered, such as CEA, CA15-3, HER2, and so on. CEA, which exists in the breast ductal secretions, showed a lower sensitivity of 58%, a lower AUC of 0.8750, and a lower DOR of 7.70 for breast cancer diagnosis (32). One study combined four biomarkers, including M-CSF, MMP-9, TIMP-1, and CA 15-3, drew a lower sensitivity, specificity, and AUC for breast cancer. The sensitivity, specificity, and AUC were 84%, 83%, and 0.9125, respectively (33).

In this study, we found a high diagnostic value of concentration of cfDNA, of which the sensitivity and specificity reached 87 and 87%, respectively. Additionally, AUC is considered as an indicator of good diagnostic performance when the value is >0.90 (34). Therefore, the value of 0.93 in our study indicated that concentration of cfDNA had a good diagnostic accuracy for breast cancer. According to the above results, we can see that compared with other biomarkers of breast cancer, whether they are novel or classical, cfDNA may have better performance on detection of breast cancer.

We also conducted subgroup analyses. The results showed there were no obvious differences among the subgroup analyses for the sensitivity, specificity, and AUC. But the DORs varied from 15.80 to 79.11. When we only incorporated the studies using plasma samples, the highest sensitivity, specificity, and AUC of cfDNA on breast cancer detection were obtained, and they were also higher than pooled sensitivity, specificity, and AUC, respectively. These results indicated that plasma may be a better source for cfDNA in detection of breast cancer.

The original studies included in our analysis showed obvious differences in sensitivity and specificity. It is widely accepted that malignancy will increase the bloodstream concentration of cfDNA. However, in some abnormal condition, such as inflammation (35), arthritis (36), trauma (37), and even after exhaustive exercise (38), the concentration of cfDNA increases too. It reminds us that benign breast lesions and other illness should be firstly excluded when we use cfDNA to detect breast cancer. Also, the diagnostic efficiency of cfDNA on breast cancer detection can be much improved in combination with other known biomarkers (CA15-3, CEA, HER2, and so on) for breast cancer.

This study also contains several limitations. Firstly, as only 13 studies were included in this meta-analysis, compounded by the small number of included studies, it may be insufficient to yield a robust result. Secondly, heterogeneity existed between the selected studies, although it was impossible to determine all sources of heterogeneity. We did not include some covariates due to the unavailable data. These probable covariates included tumor stage, metastasis condition, tumor size, the risk of breast cancer, and so on. Thirdly, subgroup analyses on several covariates were unable to perform due to the various data, such as reference gene and cut-off value. Moreover, language bias might exist due to the references being restricted to English. Even so, to our knowledge, this study is the first to systematically evaluate the diagnostic value of the concentration of cfDNA in breast cancer in the whole population and various subgroups.

This meta-analysis suggests that the concentration of cfDNA has potential first diagnostic value for breast cancer due to the high sensitivity, specificity and AUC, and it may be a potential screening tool for breast cancer. In particular, plasma may be a better source of cfDNA in detection of breast cancer. Further large-scaled, well-designed studies are required to confirm our findings, and to provide a basis for future clinical practice. Moreover, the first diagnostic value of concentration of cfDNA for breast cancer could be validated by clinical trials.

## Data Availability

All datasets generated for this study are included in the manuscript.

## Author Contributions

DY and YT made substantial contributions to conception and design, acquisition of data, analysis of data, and drafting the manuscript. XG was involved in design and drafting the manuscript. LF, ZJ, and SY were involved in drafting the manuscript and statistical analysis. JJ and YF provided support in statistical analysis. MY and HX were involved in data interpretation. LS participated in the preparation of the manuscript and provided general support. JL designed, coordinated, and supervised the study and he was involved in data analysis and interpretation and drafted the manuscript. All authors read and approved the final manuscript.

### Conflict of Interest Statement

The authors declare that the research was conducted in the absence of any commercial or financial relationships that could be construed as a potential conflict of interest.
